# Finishing pigs that are divergent in feed efficiency show small differences in intestinal functionality and structure

**DOI:** 10.1371/journal.pone.0174917

**Published:** 2017-04-05

**Authors:** Barbara U. Metzler-Zebeli, Peadar G. Lawlor, Elizabeth Magowan, Ursula M. McCormack, Tânia Curião, Manfred Hollmann, Reinhard Ertl, Jörg R. Aschenbach, Qendrim Zebeli

**Affiliations:** 1 University Clinic for Swine, Department for Farm Animals and Veterinary Public Health, University of Veterinary Medicine, Vienna, Austria; 2 Teagasc Pig Development Department, Animal & Grassland Research & Innovation Centre, Moorepark, Fermoy, Co. Cork, Ireland; 3 Agri-Food and Biosciences Institute, Agriculture Branch, Large Park, Co. Down, Hillsborough, Northern Ireland, United Kingdom; 4 Department of Science and Computing, Waterford Institute of Technology, Waterford, Co. Waterford, Ireland; 5 Institute of Animal Nutrition and Functional Plant Compounds, Department for Farm Animals and Veterinary Public Health, University of Veterinary Medicine, Vienna, Austria; 6 VetCore facility for Research, University of Veterinary Medicine, Vienna, Austria; Freie Universität Berlin, Berlin, Germany; 7 Institute of Veterinary Physiology, Freie Universität Berlin, Berlin, Germany; Universitat de Lleida, SPAIN

## Abstract

Controversial information is available regarding the feed efficiency-related variation in intestinal size, structure and functionality in pigs. The present objective was therefore to investigate the differences in visceral organ size, intestinal morphology, mucosal enzyme activity, intestinal integrity and related gene expression in low and high RFI pigs which were reared at three different geographical locations (Austria, AT; Northern Ireland, NI; Republic of Ireland, ROI) using similar protocols. Pigs (n = 369) were ranked for their RFI between days 42 and 91 postweaning and low and high RFI pigs (n = 16 from AT, n = 24 from NI, and n = 60 from ROI) were selected. Pigs were sacrificed and sampled on ~day 110 of life. In general, RFI-related variation in intestinal size, structure and function was small. Some energy saving mechanisms and enhanced digestive and absorptive capacity were indicated in low versus high RFI pigs by shorter crypts, higher duodenal lactase and maltase activity and greater mucosal permeability (P < 0.05), but differences were mainly seen in pigs from AT and to a lesser degree in pigs from ROI. Additionally, low RFI pigs from AT had more goblet cells in duodenum but fewer in jejunum compared to high RFI pigs (P < 0.05). Together with the lower expression of *TLR4* and *TNFA* in low versus high RFI pigs from AT and ROI (P < 0.05), these results might indicate differences in the innate immune response between low and high RFI pigs. Results demonstrated that the variation in the size of visceral organs and intestinal structure and functionality was greater between geographic location (local environmental factors) than between RFI ranks of pigs. In conclusion, present results support previous findings that the intestinal size, structure and functionality do not significantly contribute to variation in RFI of pigs.

## Introduction

Finding effective strategies to improve feed efficiency (**FE**) is a major goal in livestock production systems [1]. The FE is more difficult to quantify than growth and different metrics have been developed. While feed conversion ratio (**FCR**) is a simple ratio trait, the residual feed intake (**RFI**), defined as the difference between observed and expected feed intake (**FI**), accounts for component traits such as body weight (**BW**) and level of production [1]. Intensive research has advanced our understanding about the biological principles underlying diverging RFI in pigs [2–5]. One factor that may contribute to diverging RFI in pigs is the digestive efficiency as well as nutrient demands for the intestinal mucosal immune response and integrity [2–5]. The extent of intestinal nutrient uptake greatly relies on the interplay between digestive secretions, the intestinal absorptive surface and the permeability of the intestinal epithelium [6,7]. More feed efficient animals consume less feed based on their growth and maintenance requirements [8]. Since the FI substantially influences the size and energy requirement of the intestine to degrade the ingested feed [9–11], more feed efficient animals should have a smaller GIT and hence lower energy demands for basal maintenance of the GIT [2]. Moreover, the mucosal integrity is important to consider with respect to translocation of intestinal antigens, thereby triggering energetically costly immune responses and affecting growth efficiency [5,7]. Previous findings for RFI-related differences in GIT size, innate immune response and digestive efficiency in pigs were equivocal [2–5, 12,13]. Some studies reported an enhanced ileal digestibility of energy and expression of *SGLT1* and *GLUT2* in the jejunum of growing pigs [3,14], whereas other did not find RFI-related differences for digestive efficiency [2,12]. Likewise, gene expression data indicated that the ileal mucosal tight-junction-protein expression and innate immune signaling pathways may play a minor role for variation in RFI of pigs in an unchallenged condition [4,5]. However, serum acute-phase-proteins and ileal inflammation markers indicated a stronger immune response in high compared to low RFI pigs [5].

To date, most research regarding the impact of the gastrointestinal tract (**GIT**) on variation in RFI has been conducted under controlled conditions in one environment [2–4,13], whereby differences in environment, pig age, diet type and selection strategies for RFI used in the different studies may explain the controversial findings [15]. Therefore, we hypothesized that if the size, structure and functionality of the GIT play a role in variation of the RFI in pigs, similar RFI-relationships should be found in low and high RFI pigs which were raised under similar conditions in several environments.

The present objective was to investigate the differences in visceral organ size, intestinal structure, mucosal enzyme activity in duodenum, as well as jejunal mucosal integrity and gene expression of nutrient transporters, tight-junction proteins and components of the innate immune response in low and high RFI pigs which were raised at three different locations.

## Materials and methods

### Animal procedures and selection for feed efficiency

Three pig experiments were conducted at three different research facilities using similar terminal sires, feeding and management protocols, comprising the experimental design, data and sample collection: University of Veterinary Medicine Vienna (Austria; AT), AFBI Hillsborough (Northern Ireland, UK; NI), and Teagasc Moorepark (Republic of Ireland; ROI). For the pig trial in AT, all animal experimentation procedures were approved by the institutional ethics committee of the University of Veterinary Medicine (Vienna, Austria) and the national authority according to paragraph 26 of Law for Animal Experiments, Tierversuchsgesetz 2012 –TVG 2012 (GZ 68.205/0058-WF/II/3b/2014). In NI, the pig trial was conducted under project licences PPL 2751 and PPL 2781 obtained from the Department of Health, Social Services and Public Safety (DHSSPS) which adhere to the Animals (Scientific 120 Procedures) Act 1986. In the ROI, the trial was approved by the animal ethics committees of Teagasc (TAEC9/2013) and Waterford Institute of Technology (13/CLS/02) and an experimental license (number AE1932/P004) was obtained from the Irish Health Products Regulatory Authority (HPRA).

The intact litters of 36 sows (6 in AT, 8 in NI and 22 in ROI) were used in this experiment, amounting to 64, 87 and 228 piglets in AT, NI and ROI, respectively. In order to reduce the impact of the sire on FE of the progeny, sows were randomly inseminated with semen from boars (Hermitage Genetics, Kilkenny, Ireland) which had a high estimated breeding value for feed conversion efficiency. One common boar was used at all locations in combination with 3 boars being specific for AT, 3 boars specific for NI and 10 boars specific for ROI. Sows were fed similar diet formulations (gestation and lactation diets, S1 Table). Pigs were weaned at 28 days of age. Siblings were group-housed. Pigs were fed with the same sequence of diets with the same ingredient composition and chemical composition (starter, link, weaner and finisher, S1 Table) across locations via Feed Intake Recording Equipment (FIRE) feeders (Schauer Agrotonic, Wels, Austria). Water and feed were freely available throughout the nursery and fattening phases.

At each location, pigs were placed on test between day 42 and 91 postweaning when intake was recorded daily and pig weight as well as back-fat depth was recorded weekly. Average daily feed intake (ADFI) and average daily gain (ADG) measurements from day 42 to day 91 postweaning across the three locations. Following this test period (day 91 postweaning) RFI was calculated for each pig following which pigs were ranked according to their RFI. Pigs with extreme RFI were selected within litter and sex. The RFI was calculated as the residuals from a least squares regression model of ADFI on ADG, metabolic live weight, sex and all relevant two-way interactions, as well as the effects of back-fat and muscle-depth using the PROC REG procedure in SAS (version 9.4; SAS Inst. Inc., Cary, NC, USA). For extreme animals of the same sex within a litter to be selected, their RFI had to be greater than two standard deviations distant from the mean of the two RFI ranks. A total of 100 pigs (16 from AT, 24 from NI and 60 from ROI) were ranked as either low or high RFI.

### Visceral organ sampling

Pigs had free access to feed until slaughter (approximately day 110 postweaning, see Supporting Information) and were weighed before slaughter. At slaughter, the abdominal cavity was opened and the visceral organs including the gastrointestinal tract, liver, lungs, and kidneys were removed. Weight of liver, lungs and kidneys were recorded at all three locations. Length and empty weight of the small intestine and cecum were measured only at AT. In AT, the small intestine and cecum were emptied of digesta, cleaned with water or, for intestinal segments where tissue samples were taken, with phosphate-buffered saline (PBS), cleared of fat and connective tissue, blotted dry on paper towel and weighed. To account for BW differences among pigs, weight of the visceral organs and intestinal length were expressed per kg of BW.

### Histological measurements

Intestinal tissue samples for histological measurements were collected in AT and ROI, whereby duodenal, ileal and cecal tube samples were collected at both locations and jejunal tube samples only in AT. Intestinal tube samples (2–3 cm in length) were taken from the duodenum (15 cm distal from the pyloric junction), jejunum (2.5 m proximal to ileo-cecal junction) and ileum (15 cm proximal to the ileo-cecal junction) and the terminal end of the cecum. Intestinal tube pieces were washed with PBS until all digesta were removed. Tube pieces were immediately placed in neutral-buffered (pH 7.0) formalin (4% vol/vol) in AT or in No-Tox fixative (alcohol/aldehyde fixative; Cruinn Ltd., Dublin, Ireland) in ROI. After fixation in formalin for 48 h at AT, intestinal tube samples were dehydrated, cleared and embedded in paraffin. Three discontinuous paraffin-embedded 3 to 4 μm-thick sections per intestinal site and pig were routinely deparaffinized and stained with hematoxylin and eosin for determination of villus height and crypt depth, and three sections with Periodic acid Schiff stain for enumeration of goblet cells. Slides were examined on a Leica DM2000 light microscope (Leica Microsystems, Wetzlar, Germany) and digital images were captured. The villus height from the tip to the villus-crypt junction, villus width at the middle of the length of the villus, and the crypt depth from the base of the villus to the mucosa, circular and longitudinal muscular layers were measured, and goblet cells were counted per 250 μm of villus or crypt epithelium using the image analysis software ImageJ (version 1.47; National Institutes of Health, Maryland, USA). For each trait, 15 measurements were taken from intact well-oriented, crypt-villus units. Villus height and width were measured at 4-times objective magnification as well as crypt depth, muscular layers and goblet cell counts at 10-times objective magnification.

### Brush border enzyme activity analysis

At all three locations, after thoroughly cleaning the intestinal tissue of digesta and blotting dry on paper, the mucosa was scraped from a 15 cm long section of duodenum, taken distal to the sample taken for histology and distal to the pancreatic duct, using a glass slide. The scraped mucosa was immediately snap-frozen in liquid nitrogen and stored at -80°C. Preparation of duodenal homogenates (20%, w/v) and mucosal enzyme activity measurements were performed as previously described [16]. The mucosal homogenate samples were analyzed for maltase (EC 3.2.1.20), sucrase (EC3.2.1.48) and lactase (EC 3.2.1.23) activities in one gram protein by incubating mucosal homogenates at +37°C for 35 min with 250 mM maltose, 250 mM sucrose and 250 mM lactose, respectively, in 0.05 M maleate buffer (pH 6.0). The released glucose was determined using the glucose oxidase-peroxidase method and measuring absorbance at 610 nm. Enzyme activities were related to the protein concentration in the mucosal sample using a commercial Coomassie Blue dye-binding protein quantitation assay (Roti-Quant, Carl Roth; Bio-Rad Laboratories Ltd.) according to the Bradford method using bovine serum albumin as standard. All enzyme activities were expressed as micromoles of substrate hydrolysed per minute per gram of protein (U/g protein).

### Integrity of intestinal barrier

Differences in jejunal electrophysiological parameters and permeability marker flux were evaluated only at AT. A jejunal tube piece (20 cm) was taken proximal to the jejunal tissue sample taken for histological measurements and was immediately transferred into ice-cold transport buffer (S2 Table) which was pre-gassed with carbogen (95% O_2_ and 5% CO_2_). After removal of the first centimeter of the tissue piece, three successional samples from the distal 10 cm of the jejunal tube piece were evaluated in parallel. Each jejunal tissue piece was opened at the mesenterium, rinsed with transport buffer to remove remaining digesta particles, stripped of the outer serosal layers (*Tunica serosa* and *Tunica muscularis*) and mounted in one Ussing chamber. The time elapsing between the death of the pig and mounting the tissue pieces into the Ussing chambers was between 15 and 30 min. Electrophysiological measurements were taken as previously described [17]. Briefly, each replicate tissue piece (0.91 cm^2^) was bathed in experimental buffer solution (S2 Table) and gassed with carbogen (95% O_2_−5% CO_2_). The temperature was maintained at 38°C using circulating warm water. Each Ussing chamber was connected to a pair of dual channel current and voltage electrodes (Ag–AgCl) which were submerged in 3% agar bridges filled with 3 m potassium chloride. The tissue was alternatively pulsed with a positive or negative pulse of 20 μA for 100 ms of duration. After an equilibration period of 20 min under open-circuit conditions, the tissue was short-circuited by clamping the voltage to zero. The potential difference (mV), short-circuit current (I_sc_, μA/cm^2^) and transepithelial resistance (Ω × cm^2^) were continuously recorded using a microprocessor-based voltage-clamp device and software (version 9.10; Mussler, Microclamp, Aachen, Germany). The tissue conductance (G_T_, mS/cm^2^) was calculated as the reciprocal of the R_T_.

After recording electrophysiological measurements for 5 min, fluorescein 5(6)-isothiocyanate (FITC, final concentration: 0.1 mmol/L; Sigma-Aldrich, Schnelldorf, Austria) and horse-radish peroxidase (HRP, final concentration: 1.8 μmol/L; Carl Roth GmbH+Co.KG, Karlsruhe, Germany) were added to assess the mucosal to serosal flux and hence the paracellular permeability of the jejunum. Solution samples from the basolateral side were taken at 60, 120 and 180 min, whereas solution samples from the mucosal side were collected at 70 and 170 min after marker addition to measure marker flux rates. At the end of the experiment (185 min after the voltage clamp), theophylline (inhibitor of the phosphodiesterase; final concentration, 8 mm) was added to both chamber sides to monitor tissue vitality. The FITC and HRP concentrations in mucosal and serosal buffers were analyzed and mucosal-to-serosal flux rates of FITC and HRP were calculated as described in Metzler-Zebeli et al. [17].

### Candidate gene expression

Mucosa was scraped from a 30 cm length of jejunum immediately distal to the jejunal tissue sample taken for histological analysis. Total RNA was isolated from the jejunal mucosal scrapings of pigs from AT and ROI using mechanical homogenization and the RNeasy Mini Kit (Qiagen, Hilden, Germany) as recently described [18]. The RNA isolates were treated with DNase I (RNA Clean & Concentrator-5 Kit, Zymo Research, Irvine, USA). The quality of RNA was verified using the Agilent 2100 Bioanalyzer (Agilent Technologies, Santa Clara, USA). The RNA integrity numbers (RIN) of mucosa samples from AT ranged between 9 and 10, whereas mucosa samples from ROI ranged between 6 and 10. Single stranded cDNA was synthesized from 1 μg of total RNA using the High Capacity Reverse Transcription Kit (Life Technologies Foster City, USA).

Primers were designed using the Primer Express Software version 3.0 (Life Technologies; S3 Table). If possible, primer pairs were located on different exons. Candidate genes were sodium-dependent glucose transporter 1 (*SGLT1*), monocarboxylate transporter 1 (*MCT1*), intestinal alkaline phosphatase (*ALPI*), mucin 2 (*MUC2*), tight-junction proteins [zona occludens 1 (*ZO1*) and occludin (*OCLN*)], interleukin-1β (*IL1B*), tumor-necrosis-factor-α (*TNFA*), toll-like receptor 2 (*TLR2)* and 4 (*TLR4*), (see S3 Table). Five house-keeping genes (HKGs) [β-actin (*ACTB*), hypoxanthine phosphoribosyltransferase 1 (*HPRT1*), glyceraldehyde 3-phosphate dehydrogenase (*GAPDH*), β-2 microglobulin (*B2M*), small nuclear ribonucleoprotein D3 polypeptide (*SNRPD3*) and ornithine decarboxylase antizyme 1 (*OAZ1*)] were included. The expression stability of all five HKGs was assessed using the geNorm software tool [19]. The geometric mean of the two most stably expressed genes (*GAPDH*, *SNRPD3*) was used for normalization of the target gene expression levels.

Amplifications of target and HKGs were performed on a ViiA 7 Real-time PCR system (Life Technologies, Carlsbad, CA, USA) in 20 μl reactions including 25 ng cDNA template, 200 nM of each primer, 0.2 mM of each dNTP, 3 mM MgCl_2_, 1 × buffer B2 (Solis BioDyne, Tartu, Estonia), 50 nM ROX reference dye (Invitrogen, Carlsbad, USA), 0.4 × EvaGreen fluorescent dye (Biotium, Hayward, USA) and 1 unit of HOT FIREPol DNA polymerase (Solis BioDyne) [18]. All reactions were run in duplicate using the following temperature protocol: 95°C for 10 min, 40 cycles of 95°C for 15 sec and 60°C for 1 min, followed by the generation of melting curves. Reverse transcription controls (RT minus) were included in order to control for residual DNA contamination. The geometric mean expression level of the two most stably expressed HKGs (*GAPDH*, *SNRPD3*) was used for normalization of the target gene expression levels. The expression of the target gene, normalized to the mean of the two HKG, was calculated relative to the expression in the jejunum of one high RFI pig from AT using the 2^-ΔΔCt^ method [20]. Amplification efficiencies (E = 10^(-1/slope)-1^) of all primer sets are provided in S3 Table.

### Chemical analysis of diets

Nutrient analysis in diets was performed essentially as described before [21,22]. Dry matter was determined after oven-drying for 4 h at 103°C, crude ash by overnight incineration at 550°C, crude protein (nitrogen × 6.25) by the Kjeldahl method, ether extracts, crude fiber, and first-limiting amino acids [22].

### Statistical analysis

The Shapiro-Wilk test was used to test for normality of data distribution for all variables using the PROC UNIVARIATE procedure in SAS (version 9.4; SAS Inst. Inc., Cary, NC). Additionally, the Cook’s distance (Cook’s D) test was used to determine any influential observation on the model.

All variables were normally distributed and analyzed by ANOVA using the MIXED procedure in SAS. Fixed effects included in the model were sex, RFI rank, location and RFI rank × location interaction. For variables which were determined only at one location, fixed effects included only sex and RFI rank. The sow was included as random effect and pig was the experimental unit. Degrees of freedom were approximated by the method of Kenward-Roger and the covariance structure was compound symmetry. The Tukey correction for multiple testing was used for pairwise comparisons between least squares means. Least squares means were computed and significance declared at *P* ≤ 0.05. A trend was considered at 0.05 < *P* ≤ 0.10.

For variables that were available from at least two locations, Pearson’s correlation analysis (PROC CORR) in SAS was used to quantify the relationship between individual RFI and the intestinal variables, visceral organ size, intestinal morphology, mucosal enzyme activity and gene expression in the jejunum.

## Results

### Performance and feed efficiency

Differences in ADFI, ADG and RFI values between low and high RFI pigs from all locations are presented in Table 1. The RFI values of low RFI pigs from AT, NI and ROI were 2127, 407 and 2010 g lower than those of their high RFI counterparts (*P* < 0.001), respectively. The low RFI pigs from AT, NI and ROI had a 426, 402 and 238 g lower ADFI compared to the respective high RFI pigs (*P* < 0.001), whereas the ADG was similar between RFI ranks. Location affected ADFI and ADG of pigs, whereas the RFI × location interaction indicated that the high RFI value of pigs from NI was smaller than that of pigs from AT and ROI (*P* < 0.05). At slaughter, BW was similar between RFI ranks, whereby pigs from NI weighed 10 and 15 kg less than those from AT and ROI, respectively (*P* < 0.01; Table 1).

**Table 1:** Least squares means of average daily feed intake (ADFI), average daily gain (ADG) and feed efficiency of finishing pigs ranked on residual feed intake across geographic locations.

Parameter^a^	Location^d^	Low RFI	High RFI	SEM	RFI, *P*-Value^e^	Location, *P*-Value^e^	RFI × location, *P*-Value^e^
ADFI (g/day)	AT	1949	2375	101.7	<0.001	0.002	0.330
	NI	1625	2027	83.7			
	ROI	1953	2191	52.1			
ADG (g/day)	AT	1077	1133	58.0	0.44	0.006	0.670
	NI	886	906	48.6			
	ROI	943	936	31.5			
RFI (g)	AT	-956^c^	1171^a^	278.7	<0.001	0.690	0.001
	NI	-200^c^	207^b^	227.6			
	ROI	-840^c^	1170^a^	143.9			
BW at sacrifice (kg)	AT	106.4	109.9	4.141	0.229	0.009	0.724
	NI	91.0	94.3	3.927			
	ROI	102.0	102.7	1.938			

Values are least squares means ± standard error of the mean (SEM).

^a-c^For RFI, LS means with a different superscript were statistically different (P<0.05).

^d^AT, Austria; NI, Northern Ireland; ROI, Republic of Ireland. ADFI, average daily feed intake; ADG, average daily gain; BW, body weight; FE, feed efficiency; RFI, residual feed intake. ADFI, ADG and RFI were determined between days 42 and 91 postweaning.

^e^*P*: probability level of fixed effects RFI, location and their two-way interaction.

### Visceral organ weights and intestinal length

Low and high RFI pigs had similar weight of the visceral organs heart, lungs and liver (Table 2). Small intestinal and cecal length, which was only determined in AT, was also not different between low and high RFI pigs.

**Table 2:** Least squares means of visceral organ weight and intestinal length in finishing pigs ranked on residual feed intake across geographic locations.

Parameter^a^	Location^a^	Low RFI	High RFI	SEM	RFI, *P*-Value^b^	Location, *P*-Value^b^	RFI × location, *P*-Value^b^
Heart (g/kg BW)	AT	4.75	4.46	0.173	0.210	<0.001	0.168
	NI	4.34	4.20	0.152			
	ROI	3.62	3.71	0.080			
Kidney (g/kg BW)	AT	4.12	3.82	0.220	0.496	0.120	0.135
	NI	3.73	4.03	0.195			
	ROI	3.48	3.71	0.101			
Lungs (g/kg BW)	AT	8.57	7.38	0.645	0.330	0.015	0.297
	NI	5.73	6.01	0.592			
	ROI	7.40	7.30	0.301			
Liver (g/kg BW)	AT	19.61	18.85	0.773	0.708	<0.001	0.314
	NI	17.38	18.41	0.704			
	ROI	16.11	16.30	0.360			
Pancreas (g/kg BW)	AT	0.80	0.72	0.061	0.327	-	-
Small intestine (g/kg BW)	AT	18.96	18.85	0.846	0.922	-	-
Cecum (g/kg BW)	AT	1.39	1.38	0.059	0.899	-	-
Small intestinal length (cm/kg BW)	AT	14.21	14.74	1.0890	0.711	-	-
Cecum length (cm/kg BW)	AT	0.27	0.28	0.0180	0.712	-	-

Values are least squares means ± standard error of the mean (SEM).

^a^AT, Austria; NI, Northern Ireland; ROI, Republic of Ireland. FE, feed efficiency; RFI, residual feed intake.

^b^*P*: probability level of fixed effects RFI, location and their two-way interaction.

Although visceral organ weight was adjusted for BW, heart, lungs and liver weight were affected by location. Heart weight was 20 and 14% lower in pigs from ROI compared to those from AT and NI (*P* < 0.05). Lungs, in turn, were lighter in pigs from NI compared to pigs from AT and ROI, respectively (*P* < 0.05), whereas liver weight was 16% lower in pigs from ROI compared to pigs from AT (*P* < 0.05).

### Intestinal morphology

Histo-morphological parameters were measured in pigs from AT and ROI, whereby low and high RFI pigs showed RFI-related structural differences along the small intestine. Low RFI pigs tended to have 14% shorter crypts in the duodenum than high RFI pigs (Table 3), resulting in the trend for a 15% higher villus height:crypt depth ratio in low RFI pigs (*P* < 0.10). Moreover, low RFI pigs had 35% more goblets cells per villus length in the duodenum compared to high RFI pigs, whereby the location × RFI interaction (*P* < 0.05) indicated that the RFI effect was mainly detectable in pigs from AT. In contrast, low RFI pigs from AT had 49% fewer goblet cells in the jejunum than high RFI pigs (*P* < 0.01). Overall, pigs from ROI had shorter villi and deeper crypts in the duodenum but shallower crypts in the cecum, as well as fewer goblet cells in the duodenum, ileum and cecum compared to pigs from AT (*P* < 0.05).

**Table 3:** Least squares means of morphological characteristics of different intestinal segments in finishing pigs ranked on residual feed intake across geographic locations.

	AT^c^			ROI^c^					
Parameter^a^	Low RFI	High RFI	SEM	Low RFI	High RFI	SEM	RFI, *P*-Value^d^	Location, *P*-Value^d^	RFI × location, *P*-Value^d^
Duodenum									
Villus height (μm)	775	732	38.61	543	541	27.28	0.401	<0.001	0.443
Villus width (μm)	171	156	15.96	145	137	11.27	0.306	0.241	0.719
Crypt depth (μm)	323	411	49.71	522	556	35.12	0.084	0.005	0.442
Muscularis layer (μm)	737	703	46.38	-	-	-	0.585	-	-
Villus height: crypt depth	2.19	1.76	0.23	1.29	1.17	0.16	0.091	0.008	0.349
Goblet cells (per villus height)	19.4a	12.4^b^	1.550	9.7^b^	11.6^b^	1.095	0.021	0.006	<0.001
Jejunum									
Villus height (μm)	505	524	31.120	-	-	-	0.640	-	-
Villus width (μm)	167	167	8.06	-	-	-	0.991	-	-
Crypt depth (μm)	226	231	18.370	-	-	-	0.814	-	-
Muscularis layer (μm)	531	472	41.46	-	-	-	0.300	-	-
Villus height: crypt depth	2.28	2.30	0.15	-	-	-	0.910	-	-
Goblet cells (per villus height)	16.44	24.38	1.848	-	-	-	0.008	-	-
Ileum									
Villus height (μm)	446	465	28.34	414	422	12.84	0.433	0.196	0.752
Villus width (μm)	179	194	10.33	182	179	4.68	0.367	0.568	0.157
Crypt depth (μm)	242	282	32.67	275	267	14.80	0.437	0.786	0.242
Muscularis layer (μm)	880	778	53.05	-	-	-	0.169	-	-
Villus height: crypt depth	1.98	1.73	0.226	1.57	1.72	0.102	0.726	0.370	0.173
Goblet cells (per villus height)	21.07	23.93	1.868	16.43	16.38	1.313	0.282	0.008	0.266
Cecum									
Crypt depth (μm)	489	504	31.36	324	318	26.76	0.816	0.002	0.587
Goblet cells (per crypt depth)	963	1031	14.83	405	373	11.38	0.822	0.008	0.527

Values are least squares means ± standard error of the mean (SEM).

^a,b^ Mean values within a row with unlike superscript letters were significantly different (*P* < 0.05).

^c^AT, Austria; NI, Northern Ireland; ROI, Republic of Ireland. FE, feed efficiency; RFI, residual feed intake.

^d^*P*: probability level of fixed effects RFI, location and their two-way interaction.

### Mucosal disaccharidase activity in duodenum

Differences in the activity of the disaccharidases were found between RFI ranks. However, as indicated by the RFI × location interaction (lactase and maltase, *P* < 0.05; sucrase, *P* < 0.10; Table 4), these RFI-related differences mainly existed in pigs from AT but not in pigs from NI and ROI. Accordingly, low RFI pigs from AT had a 67, 34 and 40% higher lactase, maltase (*P* < 0.05) and sucrase activity (*P* < 0.10), respectively, at the duodenal mucosa than their high RFI counterparts.

**Table 4:** Least squares means of mucosal disaccharidase activities in the duodenum of finishing pigs ranked on residual feed intake across geographic locations.

Item	Location^c^	Low RFI	High RFI	SEM	RFI, *P*-Value^d^	Location, *P*-Value^d^	RFI × location, *P*-Value^d^
Lactase (U/g protein)	AT	79^a^	26^b^	10.7	<0.001	0.002	<0.001
	NI	15	22	9.0			
	ROI	27	34	9.4			
Maltase (U/g protein)	AT	803^a^	532^b^	79.9	0.332	<0.001	0.004
	NI	187	249	66.6			
	ROI	284	369	68.8			
Saccharase (U/g protein)	AT	30^A^	18^B^	5.9	0.844	0.007	0.085
	NI	3.5	7.6	4.9			
	ROI	18	23	5.1			

Values are least squares means ± standard error of the mean (SEM).

^a,b^ Mean values within a row with unlike superscript letters were significantly different (*P* < 0.05).

^A,B^ Mean values within a row with unlike superscript capital letters tended to be different (*P* < 0.10).

^c^AT, Austria; NI, Northern Ireland; ROI, Republic of Ireland. FE, feed efficiency; RFI, residual feed intake.

^d^*P*: probability level of fixed effects RFI, location and their two-way interaction.

### Candidate gene expression in the jejunum and intestinal integrity

**Fig 1:**
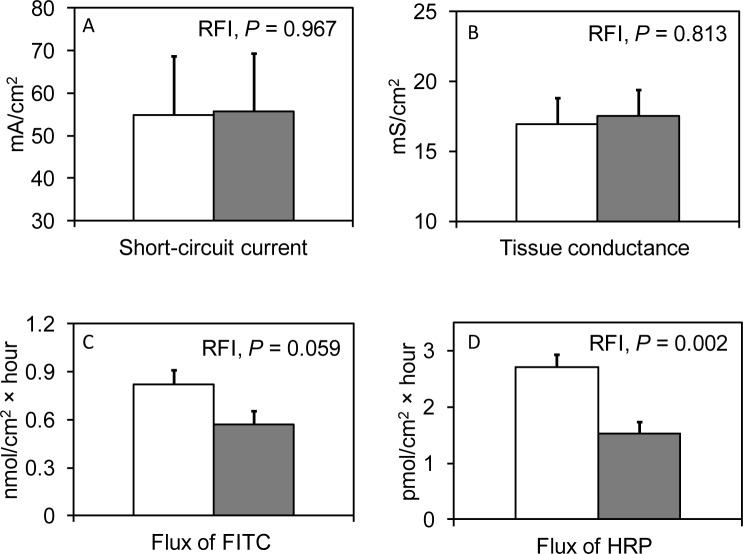
Least squares means of mucosal electrophysiological variables and mucosal-to-serosal marker flux in jejunum of finishing pigs ranked on residual feed intake (RFI). Intestinal electrophysiological variables were only determined in Austria. Results are presented as least-squares means ± SEM (□, low RFI pigs, n = 8; ■, high RFI pigs, n = 8). A) short-circuit current; B) transepithelial tissue conductance; C) mucosal-to-serosal flux of FITC; and D) mucosal-to-serosal flux of horseradish peroxidise (HRP).

Jejunal expression of nutrient transporters, such as *SGLT1*, *GLUT2* and *MCT1*was similar between RFI ranks (Table 5). With regards to innate immune signaling pathways, RFI rank affected the expression of *TLR4* with a lower expression in low RFI compared to high RFI pigs (*P* < 0.05). The trend (*P* < 0.10) for a location × RFI interaction for the expression of *TNFA* indicated a 81%-lower expression of *TNFA* in low RFI pigs from AT compared to their high RFI counterparts but no RFI-related differences in pigs from ROI. Tight-junction-protein expression was not different in low and high RFI pigs which concurred with the electrophysiological parameters (i.e., I_sc_ and G_T_) of the jejunal mucosa determined in pig from AT (Fig 1). However, the mucosal-to-serosal flux rates of FITC and HRP suggested differences in the paracellular permeability, with low RFI pigs having a 44% greater flux of HRP (*P* < 0.05) and 30% higher flux of FITC (*P* < 0.10) compared to high RFI pigs.

**Table 5:** Least squares means of mucosal expression of target genes in the distal jejunum of finishing pigs ranked on residual feed intake across geographic locations.

	AT			ROI					
Target gene	Low RFI	High RFI	SEM	Low RFI	High RFI	SEM	RFI, *P*-value^c^^d^	Location, *P*-value^d^	RFI × location, *P*-value^d^
*SGLT1*	1.77	1.84	0.455	0.92	0.94	0.370	0.857	0.198	0.940
*MCT1*	0.83	0.64	0.113	0.76	0.71	0.092	0.229	0.878	0.855
*MUC2*	0.47	0.48	0.290	0.88	1.21	0.237	0.262	0.228	0.274
*ALPI*	0.57	0.74	0.231	0.50	0.52	0.188	0.485	0.681	0.597
*TLR2*	0.59	0.46	0.368	0.53	0.38	0.299	0.980	0.690	0.510
*TLR4*	0.65	1.06	0.156	0.34	0.41	0.128	0.036	0.056	0.151
*TNFA*	0.34^b^	0.62^a^	0.122	0.28^b^	0.26^b^	0.101	0.101	0.246	0.064
*IL1B*	0.48	0.83	0.467	0.14	0.53	0.384	0.208	0.642	0.944
*OCLN*	0.75	0.64	0.113	0.74	0.71	0.092	0.475	0.159	0.807
*ZO1*	0.72	0.67	0.154	1.06	0.97	0.125	0.475	0.159	0.807

Values are least squares means ± standard error of the mean (SEM).

^a,b^ Mean values within a row with unlike superscript letters were significantly different (*P* < 0.05).

^c^AT, Austria; NI, Northern Ireland; ROI, Republic of Ireland. FE, feed efficiency; RFI, residual feed intake.

^d^*P*: probability level of fixed effects RFI, location and their two-way interaction.

### Correlation analysis

Pearson’s correlation analysis was used to correlate RFI with intestinal variables that were measured at two or all three locations (S4 Table). No significant correlations between RFI and intestinal variables were observed.

## Discussion

Characteristic features of low RFI pigs are that they eat less, spend less time eating, and have reduced physical activity [23,24]. Correspondingly in the present study, pigs selected for low and high RFI had higher ADFI and RFI values from low to high RFI across locations in the present study. Differences in pigs’ age, type of diets, management, selection strategies for RFI and breeds used may help explain discrepancies observed when the results from previous studies are compared, as these studies were limited to one batch of pigs or to a single rearing location [2–5,12,13]. In order to enhance the current knowledge about the RFI-related variation in the size, structure and functionality of the GIT in pigs, we therefore designed pig trials across three geographical locations to mirror each other as closely as possible. Current findings support earlier work [2,5,12] demonstrating that the GIT (i.e., size, structure and function) does not significantly contribute to the variation in RFI of slaughter pigs when fed a standard diet and not undergoing an immune challenge. Present results demonstrated that RFI-related variation in intestinal structure and function (i.e. duodenal activity of disaccharidases, duodenal and ileal morphology and jejunal expression of components of the innate immune system) was mostly location-specific and almost exclusively found in pigs from AT, to a lesser extent in those from ROI, and was absent in pigs from NI. Pearson’s correlation analysis confirmed the little association between pig’s RFI values and the investigated intestinal parameters across the three locations. Our results on RFI-related differences, therefore, allude to environmental factors that were intimately associated with the respective pig farm which may be more important for intestinal size, structure and functionality than pig’s RFI *per-se*.

Different sizes of visceral organs, such as the highly metabolically active GIT and liver, may have a large influence on total oxygen consumption and thus on efficiency of energy utilization [11]. Accordingly, previous results for pigs from low and high RFI lines showed an energy saving mechanism with respect to the size of the liver and empty GIT and colon weight for low RFI pigs compared to high RFI pigs [2,25]. Results from the present study, however, indicated that the visceral organ size was not an important source of variation for low and high RFI in our pigs. In fact, the environment had a greater impact on the size of liver, heart and lungs than the RFI rank of the pig. Besides the size of the GIT, the condition of the absorptive surface plays a critical role with regards to digestive and absorptive efficiency [26]. Although the intestinal villi length and crypt depth are indicative for digestive and absorptive capacity at the brush border and cell turnover, respectively [6,27], little has been reported in the literature for RFI-related variation in pigs so far. The shallower crypt depth in the duodenum of low RFI pigs in the present study may therefore suggest a reduced intestinal cell turnover rate compared to high RFI pigs and therefore may indicate an energy saving mechanism for low RFI. This effect was more obvious in pigs from AT than in pigs from ROI. Due to this RFI-related variation in the duodenal morphology, we investigated if a diverging digestive capacity was also expressed in the activity pattern of digestive enzymes in the duodenum. Since Vigors et al. [3] previously found an increased jejunal expression of sucrase-isomaltase in low versus high RFI pigs but not of proteases, we focused mainly on mucosal disaccharidases. In fact, we found a greater activity of maltase, sucrase and lactase at the duodenal brush border in low RFI pigs than in high RFI pigs but only in those from AT, although pigs from all locations were in the early postprandial state at slaughter. Vigors et al. [3] also observed a greater expression of sugar transporters *SGLT1*, *GLUT2* and fatty acid transporter *FABP2* in the jejunum together with an enhanced apparent total tract digestibility of dry matter, nitrogen and gross energy in low versus high RFI pigs. In the present study, however, we did not observe differences in the jejunal expression of nutrient transporters *SGLT1* and *MCT1* between RFI groups. In considering that Vigors et al. [3] sampled the proximal jejunum (60 cm from the stomach), whereas in the present study we collected mucosal samples from the distal part of the jejunum, this may explain the contrasting observations between the previous and the present study.

The integrity of the intestinal epithelium is not only important regarding translocation of intestinal antigens but also for nutrient uptake [7]. Similar to the findings of Mani et al. [5] who found equal tissue resistance in the ileal and colonic mucosa of growing pigs from low and high RFI lines, we did not observe RFI-related differences in jejunal electrophysiological parameters (i.e., I_sc_ and G_T_) in pigs from AT. In contrast to Mani et al. [5], however, present low RFI pigs appeared to have a greater jejunal paracellular permeability as indicated by the mucosal-to-serosal fluxes of FITC and HRP. An enhanced paracellular nutrient uptake in low RFI pigs might have compensated for the lower FI in these pigs compared to high RFI pigs. By contrast, gene expression levels of *ZO1* and *OCLN*, as important components of the tight junction protein complex [28], in the jejunum were not different between low and high RFI pigs, neither in pigs from AT nor from ROI. This finding was similar to previous gene expression results from the ileum of growing pigs from diverging RFI lines [5] and low and high RFI slaughter pigs [4]. In general, it needs to be considered that, due to translational regulation of gene expression, gene expression profiles do not entirely reflect functional protein profiles. Nevertheless, they can be regarded as the first step in the mucosal adaptive processes.

The number of goblet cells along the intestinal villi usually increases as the microbial abundance increases [29]. Microbes and microbial metabolites are recognized by the intestinal epithelium and immune cells, which leads to goblet cell differentiation [30]. In this respect, pigs from AT showed contrary RFI-related profiles in goblet cell numbers in the duodenum and jejunum, thereby indicating a higher microbial abundance in the duodenum and a lower abundance in the jejunum of low RFI pigs compared to high RFI pigs. The intestinal mucus layers secreted by goblet cells consist mainly of MUC2 mucin [30]. Despite the different goblet cell numbers in low and high RFI pigs, jejunal *MUC2* expression was equal between RFI ranks. Likewise, Vigors et al. [4] found an equal expression of the mucin genes *MUC2* and *MUC4* as well as of other genes related to the mucosal innate immune response such as TLR and pro- and regulatory cytokines in the ileum of low and high RFI pigs. In the present study, low RFI pigs had a lower expression of *TLR4* and *TNFA* in the jejunum than high RFI pigs, with the effect being stronger in pigs from AT than in those from ROI. The question arises if these observations suggested a greater mucosal abundance of gram-negative bacteria in high RFI pigs or a down-regulated mucosal innate immune signaling in low RFI pigs. As a pathogen-recognition-receptor, the TLR-4 recognizes lipopolysaccharides on the cell wall of gram-negative bacteria [31] which leads to the release of cytokines from dendritic cells, particularly TNF-α and interleukin-1. This, in turn, coordinates the influx of immune cells into the lamina propria, thereby reinforcing the mucosal barrier [31].

The observed differences in visceral organ size, intestinal structure and functionality across the three locations can be mostly related to pig farm-specific factors such as the origin of the sows, microbes encountered in the housing facilities, handling and dietary composition [32–34]. Although we used the same dietary formulation and adjusted for differences in the major nutrients (e.g., protein content and amino acid profiles and crude fiber content) in the local raw feedstuffs and used the same vitamin-mineral-premix, we did not correct for differences in the dietary fiber fractions and fatty acid composition. Although differences in these fractions should have been very subtle in the present study, these may have modified structural and functional components of the intestinal barrier function, innate immune signaling pathways and microbiota composition [35–37]. Moreover, the microbes encountered in the wider environment (e.g., sow feces, personnel, housing, water and diet) certainly differed among the three locations [38] which could have contributed to the variation in the activities of brush-border enzymes, cytokine expression, and gene expression related to nutrient absorption and mucosal barrier function [39] across locations. Results for the bacterial composition in feces (day 42 and 105 postweaning) and in ileal and cecal digesta (at slaughter) of the low and high RFI pigs, for instance, largely support different bacterial colonization among locations [38].

In conclusion, the current results support previous research that GIT structure and functionality does not significantly contribute to variation in RFI of pigs. Rather, results demonstrated that the impact of geographic location (local environmental factors) on the size of visceral organs and intestinal structure and functionality was greater than that of the RFI rank of the pig. RFI-associated differences in intestinal structure and functionality were few and mostly observed in pigs from AT, less pronounced in pigs from ROI and absent in pigs from NI. The observed RFI-associated differences, included energy saving mechanisms, such as shallower crypts in the duodenum, and enhanced digestive and absorptive capacity, as indicated by greater duodenal disaccharidase activity and jejunal paracellular permeability. Moreover, RFI-associated variation in duodenal and jejunal goblet cells and jejunal *TLR4* and *TNFA* expression may indicate different innate immune signaling in the small intestine.

## Supporting information

S1 File Dates of birth, weaning, day 42 and 91 postweaning and slaughter for pigs across locations. (DOCX)

S1 Table Dietary ingredients and chemical composition of diets (on as-fed basis). (DOCX)

S2 Table Composition of buffers used in Ussing Chamber experiment in AT. (DOCX)

S3 Table Oligonucleotide primers for target and housekeeping genes. (DOCX)

S4 Table Pearson’s correlations between residual feed intake and visceral organ size, duodenal disaccharidase activity and jejunal gene expression in pigs of low and high RFI across locations. (DOCX)
