# Supplementary material for: Finishing pigs that are divergent in feed efficiency show small differences in intestinal functionality and structure
Source: PLoS One. 2017 Apr 5;12(4):e0174917. doi: 10.1371/journal.pone.0174917 (PMC5381887; doi:10.1371/journal.pone.0174917)
Supplement: S1 File — (DOCX) [file pone.0174917.s001.docx]

**Supporting Information - Metzler-Zebeli et al.**

**S1 File. Dates of birth, weaning, day 42 and 91 postweaning and slaughter for pigs across locations**

**Republic of Ireland (ROI), replicate batch 1:**

Born: 03-04^th^ of October 2013

Weaned: 30^th^ of October 2013

Day 42 postweaning: 11^th^ of December 2013

Day 91 postweaning: 29^th^ of January 2014

Slaughter: 18^th^ of February 2014

Pigs were 111 days postweaning or 137/138 days of age when slaughtered.

**Republic of Ireland (ROI), replicate batch 2:**

Born: 21-24^th^ of September 2014

Weaned: 22^nd^ of October 2014

Day 42 postweaning: 03^rd^ of December 2014

Day 91 postweaning: 21^st^ of January 2015

Slaughter: 03^rd^ and 10^th^ of February 2014

Pigs were 104/111 days postweaning or 135/142 days of age when slaughtered.

**Austria (AT):**

Born: 11-15^th^ of April 2014

Weaned: 08^th^ of May 2014

Day 42 postweaning: 18^th^ of June 2014

Day 91 postweaning: 07^th^ of August 2014

Slaughter: 25^th^ of August 2014

Pigs were 109-113 days postweaning or 137-141 days of age when slaughtered.

**Northern Ireland (NI):**

Born: 05-07^th^ of February 2014

Weaned: 06^th^ of March 2014

Day 42 postweaning: 17^th^ of April 2014

Day 91 postweaning: 14^th^ of May 2014

Slaughter: 24^th^ of June 2014

Pigs were 110 days postweaning or 139/140 days of age when slaughtered.
