# Supplementary material for: Finishing pigs that are divergent in feed efficiency show small differences in intestinal functionality and structure
Source: PLoS One. 2017 Apr 5;12(4):e0174917. doi: 10.1371/journal.pone.0174917 (PMC5381887; doi:10.1371/journal.pone.0174917)
Supplement: S1 Table — (DOCX) [file pone.0174917.s002.docx]

**Supporting Information - Metzler-Zebeli et al.**

**S1 Table. Dietary ingredients and chemical composition of diets (on as-fed basis).**

| **Diet Type** | **Starter** | **Link** | **Weaner** | **Finisher** | **Pregnant sow** | **Lactating sow** | |
| --- | --- | --- | --- | --- | --- | --- | --- |
| Barley | - | - | 248.0 | 385.4 | 897.4 | 349.5 | |
| Wheat | 220.0 | 399.0 | 431.4 | 404.0 | - | 432.4 | |
| Maize | 80.0 | - | - | - | - | - | |
| Soya | 163.5 | 229.2 | 200.0 | 175.0 | 70.0 | 150.0 | |
| Full fat soya | 100.0 | 70.0 | 50.0 | - | - | - | |
| Lactofeed 70^a^ | 200.0 | 200.0 | - | - | - | - | |
| Skim milk powder | 125.0 | 50.0 | - | - | - | - | |
| Soya oil | 78.1 | 25.0 | 40.0 | 10.0 | 10.0 | 40.0 | |
| Lysine HCl (78.8) | 4.73 | 3.70 | 4.6 | 4.0 | 1.0 | 3.5 | |
| DL-Methionine | 3.22 | 2.33 | 1.7 | 1.0 | - | 1.0 | |
| L-Threonine (98) | 2.41 | 1.62 | 2.0 | 1.5 | - | 1.0 | |
| L-Tryptophan | 0.95 | 0.54 | 0.2 | 0.0 | - | - | |
| Vitamin and mineral mix | 3.0^b^ | 3.0^b^ | 3.0^b^ | 1.0^c^ | 1.5^d^ | 1.5^d^ | |
| Natuphos 5000 FTU/g^e^ | 0.10 | 0.10 | 0.1 | 0.1 | 0.1 | 0.1 | |
| Salt feed grade | 3.00 | 3.00 | 3.0 | 3.0 | 4.0 | 4.0 | |
| Dicalcium phosphate | 5.00 | 1.52 | 5.0 | 2.0 | 5.0 | 5.0 | |
| Limestone flour | 11.00 | 11.0 | 11.0 | 13.0 | 11.0 | 12.0 | |
|  |  |  |  |  |  |  | |
| **Chemical analysis (g/kg dry matter)** | | | | | | |  |
| Crude protein | 235.9 | 252.8 | 211.0 | 205.4 | 195.7 | 172.1 |  |
| Crude fibre | 19.8 | 23.4 | 34.8 | 37.5 | 38.9 | 31.7 |  |
| Crude ash | 66.2 | 63.5 | 48.3 | 45.4 | 44.6 | 49.8 |  |
| Ether extract | 114.7 | 57.9 | 70.7 | 27.2 | 33.2 | 63.4 |  |
| Digestible energy (MJ/kg)^f^ | 17.9 | 17.0 | 16.9 | 16.0 | 15.9 | 16.3 |  |
| Net energy (MJ/kg)^f^ | 11.4 | 10.3 | 10.6 | 9.8 | 9.5 | 10.5 |  |
|  |  |  |  |  |  |  |  |
| Amino acids (g/kg dry matter) | |  |  |  |  |  |  |
| Lysine | 16.2 | 15.0 | 13.0 | 11.1 | 6.4 | 9.9 |  |
| Methionine | 6.8 | 5.7 | 4.5 | 3.6 | 2.1 | 3.4 |  |
| Methionine + cysteine | 9.7 | 9.0 | 7.9 | 6.8 | 4.7 | 6.4 |  |
| Threonine | 10.5 | 9.8 | 8.7 | 7.5 | 4.5 | 6.5 |  |
| Tryptophan | 3.6 | 3.3 | 2.6 | 2.2 | 1.6 | 2.0 |  |

^a^Lactofeed 70 contains 70% lactose, 11.5% protein, 0.5% oil, 7.5% ash and 0.5% fibre (Volac, Cambridge, UK)*.*

^b^Premix provided per kg of complete diet: Cu, 155 mg; Fe, 90 mg; Mn, 47 mg; Zn, 120 mg, I, 0.6 mg; Se, 0.3 mg; vitamin A, 6000 IU; vitamin D_3,_ 1000 IU; vitamin E, 100 IU; vitamin K, 4 mg; vitamin B_12,_ 15 μg; riboflavin, 2 mg; nicotinic acid, 12 mg; pantothenic acid, 10 mg; choline chloride, 250 mg; vitamin B_1,_ 2 mg; vitamin B_6,_ 3 mg; Endox, 60 g.

^c^Premix provided per kg of complete diet: Cu, 15 mg; Fe, 24 mg; Mn, 31 mg; Zn, 80 mg, I, 0.3 mg; Se, 0.2 mg; vitamin A, 2000 IU; vitamin D_3,_ 500 IU; vitamin E, 40 IU; vitamin K, 4 mg; vitamin B_12,_ 15 μg; riboflavin, 2 mg; nicotinic acid, 12 mg; pantothenic acid, 10 mg; vitamin B_1,_ 2 mg; vitamin B_6,_ 3 mg.

^d^Premix provided per kg of complete diet: Cu, 15 mg; Fe, 70 mg; Mn, 62 mg; Zn, 80 mg, I, 0.6 mg; Se, 0.2 mg; vitamin A, 1000 IU; vitamin D_3,_ 1000 IU; vitamin E, 100 IU; vitamin K, 2 mg; vitamin B_12,_ 15 μg; riboflavin, 5 mg; nicotinic acid, 12 mg; pantothenic acid, 10 mg; choline chloride, 500 mg; Biotin, 200 mg; Folic acid, 5 g; vitamin B_1,_ 2 mg; vitamin B_6,_ 3 mg.

^e^Phytase; 5000 FTU/g equal to 500 FTU per kg finished feed.

^f^Digestible energy and net energy were calculated from book values.
