# Supplementary material for: Finishing pigs that are divergent in feed efficiency show small differences in intestinal functionality and structure
Source: PLoS One. 2017 Apr 5;12(4):e0174917. doi: 10.1371/journal.pone.0174917 (PMC5381887; doi:10.1371/journal.pone.0174917)
Supplement: S2 Table — (DOCX) [file pone.0174917.s003.docx]

**Supporting Information - Metzler-Zebeli et al.**

**S2 Table. Composition of buffers used in Ussing Chamber experiment in AT.**

| Item^a^ (mmol/l) | Transport buffer | Mucosal buffer | Serosal buffer |
| --- | --- | --- | --- |
| CaCl_2_.2H_2_O | 1.2 | 1.2 | 1.2 |
| MgCl_2_.6H_2_O | 1.2 | 1.2 | 1.2 |
| Na_2_HPO_4_.2H_2_O | 2.4 | 2.4 | 2.4 |
| NaH_2_PO_4_.H_2_O | 0.4 | 0.4 | 0.4 |
| KCl | 5.0 | 5.0 | 5.0 |
| NaHCO_3_ | 25.0 | 25.0 | 25.0 |
| NaCl | 115.0 | 115.0 | 115.0 |
| Mannitol | - | 12.0 | 2.0 |
| D-Glucose | 5.0 | - | 10.0 |
| HEPES | - | 5.0 | 5.0 |
| Kanamycin sulfate | - | 0.172 | 0.172 |

^a^Chemical were purchased from Sigma-Aldrich (Schnelldorf, Austria)
