# Supplementary material for: Finishing pigs that are divergent in feed efficiency show small differences in intestinal functionality and structure
Source: PLoS One. 2017 Apr 5;12(4):e0174917. doi: 10.1371/journal.pone.0174917 (PMC5381887; doi:10.1371/journal.pone.0174917)
Supplement: S3 Table — (DOCX) [file pone.0174917.s004.docx]

**Supporting Information - Metzler-Zebeli et al.**

**S3 Table. Oligonucleotide primers for target and housekeeping genes.**

| Gene symbol^a^ | Accession number^b^ | Gene name | Forward primer (5'-3') | Reverse primer (5'-3') | Amplicon size (bp) | Eff. (%)^c^ | Corr. ^d^ | Ref.^e^ |
| --- | --- | --- | --- | --- | --- | --- | --- | --- |
| *ACTB* | XM_003357928.2 | Beta-actin | GGGCATCCTGACCCTCAAG | TGTAGAAGGTGTGATGCCAGATCT | 89 | 95.4 | 0.998 | 1 |
| *B2M* | NM_213978.1 | Beta-2-microglobulin | CCCCCGAAGGTTCAGGTT | GCAGTTCAGGTAATTTGGCTTTC | 66 | 103.5 | 0.998 | 1 |
| *GAPDH* | NM_001206359.1 | Glyceraldehyde-3-phosphate dehydrogenase | GGCGTGAACCATGAGAAGTATG | GGTGCAGGAGGCATTGCT | 60 | 105.4 | 0.999 | 1 |
| *HPRT1* | NM_001032376.2 | Hypoxanthine guanine phosphoribosyl transferase | AGAAAAGTAAGCAGTCAGTTTCATATCAGT | ATCTGAACAAGAGAGAAAATACAGTCAATAG | 131 | 99.9 | 0.998 | 1 |
| *OAZ1* | NM_001122994.2 | Ornithine decarboxylase antizyme 1 | TCGGCTGAATGTAACAGAGGAA | GAGCCTGGATTGGACGTTTAAA | 70 | 99.0 | 0.998 | 1 |
| *SNRPD3* | XM_001489060.5 | Small nuclear ribonucleoprotein D3 polypeptide 18kDa | ACGCACCTATGTTAAAGAGCATG | CACGTCCCATTCCACGTC | 120 | 92.3 | 0.999 | - |
| *OCLN* | NM_001163647.2 | Occludin | TTGTGGGACAAGGAACGTATTTA | TGCCTGCCGACACGTTT | 76 | 92.0 | 0.997 | 1 |
| *ZO1* | XM_013993251.1 | Zona occludin 1 | AAGCCCTAAGTTCAATCACAATCT | ATCAAACTCAGGAGGCGGC | 131 | 101.7 | 0.998 | 1 |
| *SGLT1 (SLC5A1)* | NM_001164021.1 | Sodium-dependent glucose transporter 1 | TGTCTTCCTCATGGTGCCAA | AGGAGGGTCTCAGGCCAAA | 149 | 101.7 | 0.999 | 1 |
| *TRL2* | NM_213761.1 | Toll-like receptor 2 | AATAAGTTGAAGACGCTCCCAGAT | GTTGCTCCTTAGAGAAAGTATTGATCGT | 97 | 93.1 | 0.999 | 1 |
| *TRL4* | AB188301.2 | Toll-like receptor 4 | TGTGGCCATCGCTGCTAAC | GGTCTGGGCAATCTCATACTCA | 124 | 95.4 | 0.998 | 1 |
| *IL1B* | NM_001005149.1, NM_214055.1 | Interleukin 1, beta | AACTGGTACATCAGCACCTCTCAA | TCTTGGCGGCCTTTGGA | 71 | 95.4 | 0.998 | 1 |
| *TNFA* | EU682384.1, NM_214022.1 | Tumor necrosis factor alpha | CAGCTGGAGAAGGATGATCGA | CCAGATTCAGCAAAGTCCAGATAG | 71 | 100.6 | 0.998 | 1 |
| *MCT1 (SLC16A1)* | AM286425.1 | Monocarboxylate transporter 1 | GGTGGAGGTCCTATCAGCAG | AAGCAGCCGCCAATAATCAT | 74 | 94.4 | 0.998 | 1 |
| *ALPI* | XM_003133729.3 | Intestinal alkaline phosphatase | AGGAACCCAGAGGGACCATTC | CACAGTGGCTGAGGGACTTAGG | 83 | 99.9 | 0.999 | 2 |

^a^Alternate gene names are shown in brackets.

^b^National Center for Biotechnology Information (NCBI) Entrez Gene (http://www.ncbi.nlm.nih.gov/sites/entrez?db=gene).

^c^Eff, PCR efficiency: E = 10^(-1/slope)^-1.

^d^Corr, Correlation coefficient of standard curve.

^e^Ref, references for oligonucleotide primer sequences. 1) Metzler-Zebeli BU, Mann E, Ertl R, Schmitz-Esser S, Wagner M, Klein D, Ritzmann M, Zebeli Q. Dietary calcium concentration and cereals differentially affect mineral balance and tight junction proteins expression in jejunum of weaned pigs. Br J Nutr. 2015; 113(7):1019-31. doi: 10.1017/S0007114515000380.; 2) Metzler-Zebeli BU, Ertl R, Grüll D, Molnar T, Zebeli Q. Enzymatically modified starch up-regulates expression of incretins and sodium-coupled monocarboxylate transporter in jejunum of growing pigs. Animal, 2016; forthcoming.
