# Supplementary material for: Finishing pigs that are divergent in feed efficiency show small differences in intestinal functionality and structure
Source: PLoS One. 2017 Apr 5;12(4):e0174917. doi: 10.1371/journal.pone.0174917 (PMC5381887; doi:10.1371/journal.pone.0174917)
Supplement: S4 Table — (DOCX) [file pone.0174917.s005.docx]

**Supporting Information - Metzler-Zebeli et al.**

**S4 Table. Pearson’s correlations between residual feed intake and visceral organ size, duodenal disaccharidase activity and jejunal gene expression in pigs of low and high RFI across locations.**

| Item |  | RFI |
| --- | --- | --- |
| Heart weight (g/kg BW) | r | 0.00 |
|  | *P*-value | 1.00 |
| Kidney weight (g/kg BW) | r | 0.08 |
|  | *P*-value | 0.38 |
| Liver weight (g/kg BW) | r | 0.03 |
|  | *P*-value | 0.75 |
| Lung weight (g/kg BW) | r | 0.05 |
|  | *P*-value | 0.59 |
| Duodenal lactase (U/g protein) | r | 0.02 |
|  | *P*-value | 0.87 |
| Duodenal maltase (U/g protein) | r | 0.05 |
|  | *P*-value | 0.73 |
| Duodenal sucrase (U/g protein) | r | 0.16 |
|  | *P*-value | 0.22 |
| Jejunal *SGLT1* expression | r | -0.11 |
|  | *P*-value | 0.53 |
| Jejunal *MCT1* expression | r | 0.20 |
|  | *P*-value | 0.25 |
| Jejunal *OCLN* expression | r | 0.08 |
|  | *P*-value | 0.64 |
| Jejunal *ZO1* expression | r | 0.08 |
|  | *P*-value | 0.64 |
| Jejunal *TLR2* expression | r | -0.10 |
|  | *P*-value | 0.55 |
| Jejunal *TLR4* expression | r | -0.01 |
|  | *P*-value | 0.97 |
| Jejunal *TNFA* expression | r | 0.05 |
|  | *P*-value | 0.79 |
| Jejunal *IL1B* expression | r | 0.23 |
|  | *P*-value | 0.19 |
| Jejunal *MUC2* expression | r | 0.04 |
|  | *P*-value | 0.83 |
| Jejunal *ALPI* expression | r | -0.10 |
|  | *P*-value | 0.57 |

^a^Visceral organ weight and duodenal disaccharidase activity were measured in pigs from AT, NI and ROI; relative gene expression in jejunum was measured in pigs from AT and ROI.
